# How to Count Bugs: A Method to Estimate the Most Probable Absolute Population Density and Its Statistical Bounds from a Single Trap Catch

**DOI:** 10.3390/insects12100932

**Published:** 2021-10-13

**Authors:** Ksenia S. Onufrieva, Alexey V. Onufriev

**Affiliations:** 1 Department of Entomology, Virginia Tech, Blacksburg, VA 24061, USA; 2 College of Agriculture and Life Sciences Center for Advanced Innovation in Agriculture, Virginia Tech, Blacksburg, VA 24061, USA; 3 Department of Computer Science, Virginia Tech, Blacksburg, VA 24061, USA; alexey@cs.vt.edu; 4 Department of Physics, Virginia Tech, Blacksburg, VA 24061, USA; 5 Center for Soft Matter and Biological Physics, Virginia Tech, Blacksburg, VA 24061, USA

**Keywords:** traps, absolute population density, population ecology, IPM, conservation

## Abstract

**Simple Summary:**

The importance of conservation and pest management programs cannot be overstated as climate change, loss of biodiversity, and biological invasions are on the rise. Such programs often rely on traps for population detection and monitoring, assigning management and conservation tactics, and evaluating treatment efficacies. In this paper, we propose a universal method for any insect trap system to estimate the most probable absolute population density and its statistical bounds from a single trap catch. This approach will help take insect detection and monitoring to a new, rigorously quantitative level.

**Abstract:**

Knowledge of insect population density is crucial for establishing management and conservation tactics and evaluating treatment efficacies. Here, we propose a simple and universal method for estimating the most probable absolute population density and its statistical bounds. The method is based on a novel relationship between experimentally measurable characteristics of insect trap systems and the probability to catch an insect located a given distance away from the trap. The generality of the proposed relationship is tested using 10 distinct trapping datasets collected for insects from 5 different orders and using major trapping methods, i.e., chemical-baited traps and light. For all datasets, the relationship faithfully ($\bar{R}=0.91)$ describes the experiment. The proposed approach will take insect detection and monitoring to a new, rigorously quantitative level. It will improve conservation and management, while driv-ing future basic and applied research in population and chemical ecology.

## 1. Introduction

Traps are crucial for monitoring insect activity and are widely used in pest detection and management programs [1,2,3,4,5,6] for evaluating biodiversity and planning conservation [7,8,9] and research [10,11,12,13,14] efforts. Therefore, extensive research has been conducted to evaluate trap efficiency [15,16,17,18,19,20,21,22], estimate the range of attraction [23,24,25,26,27,28,29] and determine its probability [30,31], better interpret trap catches, and relate them to the absolute population density [32,33,34,35,36,37,38]. A correlation between trap catches and subsequent egg mass [35,36,39,40] and larval [41,42,43] density was shown for several insect trap systems; however, translating trap catches into absolute population density and, especially, interpreting zero catches continue to be a challenge [3,15,32,44].

Miller et al. [45] pioneered a method of estimating plume reach and absolute population density from catches in pheromone-baited traps [38,45]. However, this method [45] was not ideal when applied to *Lymantria dispar* (L.) (R = 0.5) [46], which led to development of a different, simple, yet mathematically rigorous, method for connecting the actual trap catch with the most probable population density, along with statistical bounds of the absolute population density [46]. Still, the key question remained unanswered: is there a general relationship of this type that might apply to all insects and trap types? In this paper, we demonstrate the generality of a simple mathematical relationship between catch probability and distance to the trap for several species of insects from various orders and two major trapping methods, i.e., chemical-baited traps and light attraction. We show how key characteristics of the population density are derived from the trapping data and describe a procedure for data collection and analysis.

Insects are the most diverse group of organisms: one may rightfully assume that it is highly improbable that their behavior with respect to attractants could be described by a universal mathematical law. However, if such a law were to be found, it could have a significant impact on the entire fields of entomology and ecology. It would make it possible to reduce the complexity of insect behavior with respect to various traps and attractants to a few parameters that could be used in the same equation. This work is about finding and validating such a law.

## 2. Materials and Methods

### 2.1. Data Collection

We searched the literature for data on insect catches in traps located at various distances from the insect release points to identify experimental datasets that satisfied the following conditions: (1) converged catch (meaning that the catch value did not increase substantially with increased trapping time, as defined in [46]) was reported for at least 4 distances between a trap and a release point, (2) number of insects released at large distance was the same or larger than at short distances, (3) no zero catch data points were reported between non-zero points. This search yielded 9 datasets: on brown marmorated stink bug (*Halyomorpha halys*) [33], codling moth (*Cydia pomonella*) [47], European pine sawfly (*Neodiprion sertifer*) [44], spotted wing drosophila (*Drosophila suzukii*) [34], Western corn rootworm (*Diabrotica virgifera*) [48], Douglas fir beetle (*Dendroctonus pseudotsugae*) [49], Southern pine beetle (*Dendroctonus frontalis*) [37], macro-moths of the families Erebidae [50] and Sphingidae [51].

### 2.2. Analysis

To the best of our knowledge, the absolute insect population density ρ(x,y) cannot be measured empirically. What is commonly measured is the number *M* of insects caught in a trap over the time interval of the experiment. Mathematically, the average $\bar{M}$ is connected, exactly, to ρ(x,y) via an integral [37] over the trap collection area of radius *R_max_*:

(1) $$\bar{M}=\iint spT_{fer}\left(r\right)\rho \left(x,y\right)dxdy=2\pi \int_{0}^{R_{max}}spT_{fer}\left(r\right)\rho \left(r\right)rdr$$

where $spT_{fer}\left(r\right)$ is the probability of catching an insect located at a distance *r* from the trap. Thus, if $spT_{fer}\left(r\right)$ is known, the average population density can be estimated from the measured $\bar{M}$ by inverting the above equation. Here, we propose that the universal relationship (Equation (2)) holds for all insect trap systems:

(2) $$spT_{fer}\left(r\right)=\{\begin{array}{c} \frac{spT_{fer}\left(0\right)}{1+{\left(\frac{r}{D_{50}}\right)}^{2}},\;r\leq R_{max}\\ 0,\;r>R_{max} \end{array}$$

where *spT_fer_*(0) [52] is the probability of catching an insect located in the immediate proximity of a baited trap, and *D_50_* is the distance from the baited trap at which the probability to catch an insect is one-half of the probability to catch an insect in the immediate proximity of the trap (*spT_fer_*(0)). Critically, the trapping time interval is not present explicitly in Equation (2) because it operates with converged catch (e.g., 3 days for *L. dispar*). Converged catch is estimated from release–recapture experiments [53], in which insects are released, and the traps are checked periodically following the release; the catch in these experiments is assumed to be converged when it stops increasing with increased trapping time. Thus, the resulting estimate of the absolute population density is an average population density over the converged catch time interval.

Equation (2) was originally developed for *L. dispar* [46] and assumed a specific pheromone-baited trapping method. Here, we generalize it to all trap types, including light, and investigate if it could be applied to other insects. We stress that this predictive relationship was derived based on general assumptions and did not involve fitting to the experimental data.

For *L. dispar*, a wealth of data points is available [46], which allowed us to come up with the most robust protocol for fitting Equation (2). Specifically, for L. dispar, males were released at distances of 0, 15, 25, 30, 45, 50, 60, 75, 80, 100, 150, 200, 250, 300, 500, 600, 900, 1000, 1200, and 1500 m from pheromone-baited traps; therefore, short and long distances were balanced and had equal weight in the determination of *D_50_*. The *L. dispar* dataset is also unique in that 12 distinct points are available for large values of r, that is significantly larger than *D_50_* (r > 75 m for *L. dispar*). The availability of multiple data points at long distances had previously allowed us [46] to come up with what we believe is the most accurate estimate of *D_50_* = 26 ± 3 m, which was based on a log–log fit for long-distance data points only. However, data available for the other insects studied here do not include *spT_fer_*(0), and the experimental design is often unbalanced: the available data points are few, and mostly for either short or long distances, but not for both. To mitigate these limitations, we developed a 2-step protocol for fitting Equation (2) to data missing *spT_fer_*(0). Step 1: Use untransformed data to estimate *spT_fer_*(0) by fitting Equation (2) to the experimental data points (we employed JMP^®^ Pro 15, SAS Institute, 2019). Step 2: Use *spT_fer_*(0) from Step 1 in Equation (3) to estimate *D_50_* by fitting Equation (3) to the log-transformed experimental data points [54]. This 2-step procedure ensures that the catches at large distances are given equal weight as the catches at short distances. For insect data that include experimentally measured *spT_fer_*(0), only step 2 should be used.

(3) $$lnspT_{fer}\left(r\right)=ln\left(\frac{spT_{fer}\left(0\right)}{1+{\left(\frac{r}{D_{50}}\right)}^{2}}\right)$$

**Figure 1:**
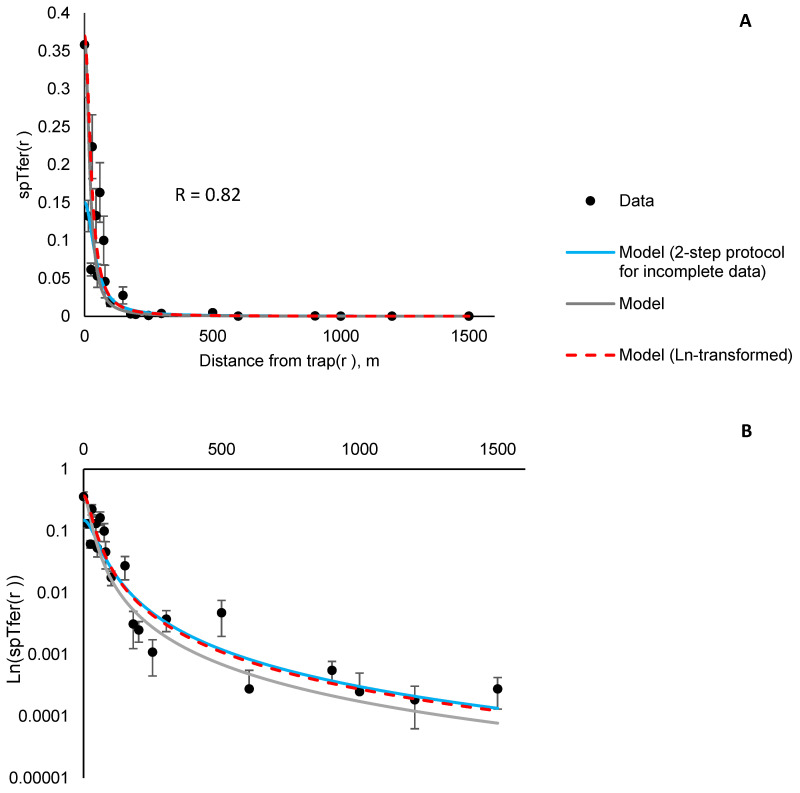
Proportion of *L. dispar* males caught in pheromone-baited traps placed at various distances from the release point (±SEM). Error bar is not shown when smaller than the symbol size. Panel (**A**), *spT_fer_(r)* vs. r illustrates the quality of the fit to Equation (2) at all distances; panel (**B**), *ln(spT_fer_(r))* vs. r illustrates the fit quality at great distances from the pheromone-baited trap.

We tested this protocol for *L. dispar* and estimated *spT_fer_*(0) = 0.15 and *D_50_* = 45 ± 5 m. This *spT_fer_*(0) is lower than the actual experimental *spT_fer_*(0)*=0.37* observed in the field [46]. Using the actual *spT_fer_*(0) in untransformed and log-transformed model, yielded *D_50_* = 21.7 ± 3 m and *D_50_* = 27.3 ± 3 m, respectively. The latter value is closest to the one obtained previously, which supports the use of the 2-step fitting procedure including the log-transformed 2nd step. The estimate of *D_50_* obtained using the 2-step protocol proposed for datasets missing *spT_fer_*(0) is higher than the estimates obtained using the other two methods; nevertheless Equation (2) with the respective parameter sets approximates the experimental data reasonably well (Figure 1) in all three cases.

Both the probability of catch in the immediate proximity to the trap, *spT_fer_*(0), and *D_50_* are crucial for establishing a relationship between catch probability and distance to a baited trap, deriving bounds for absolute population density, and estimating the most probable population density of an insect. Step-by-step instructions are available in the Supplementary Materials, the JMP scripts, and an Excel file to automatically calculate *spT_fer_*(0), *D_50_*, µ, and the most probable density ($\bar{\rho_{mp}}$) and its bounds from trap catch data are also provided (https://doi.org/10.7294/BE34-ZS61 (accessed on 10 October 2021)).

To derive bounds of the average population density $\bar{\rho }$, we used the procedure described by Onufrieva et al. [46]. Once *spT_fer_*(0), *D_50_*, and *R_max_* are estimated, we define:

(4) $$µ=\left(\frac{1}{spT_{fer}\left(0\right)}\right)\times \frac{1}{\pi D_{50}{}^{2}ln\left(1+{\left(\frac{R_{max}}{D_{50}}\right)}^{2}\right)}$$

With that, the lower and upper bounds for the average density $\bar{\rho }$ are

(5) $$\frac{\mu }{2}\;\chi^{2}\;\left(\frac{1-p}{2};2M\right)\leq \;\bar{\rho }\;\leq \frac{\mu }{2}\;\chi^{2}\;\left(1-\frac{1-p}{2};2M+2\right)$$

where M is the number of insects caught, p is the confidence level (p = 0.95 here), and $\chi^{2}\left(q;n\right)$ is the quantile function (corresponding to a lower tail area *q*) of the χ^2^ distribution with *n* degrees of freedom (Table S1).

The most probable average male density in the trapping area is

(6) $$\bar{\rho_{mp}}=µM$$

To convert the male density to the number of males per ha, assuming *D_50_* and *R_max_* are given in meters, μ in Equations (5) and (6) needs to be multiplied by 10,000 (the conversion is performed automatically in the scripts provided). Note that the most probable density and its bounds are sensitive to the values of *spT_fer_*(0) and, especially, *D_50_*, emphasizing the need for high-quality experimental data points and robust procedures to extract *spT_fer_*(0), and, especially, *D_50_* from the data. In contrast, the dependence of insect density characteristics on *R_max_* (via Equation (4)) is weak and logarithmic, which means that, in practice, a rough estimate of *R_max_* should suffice.

**Figure 2:**
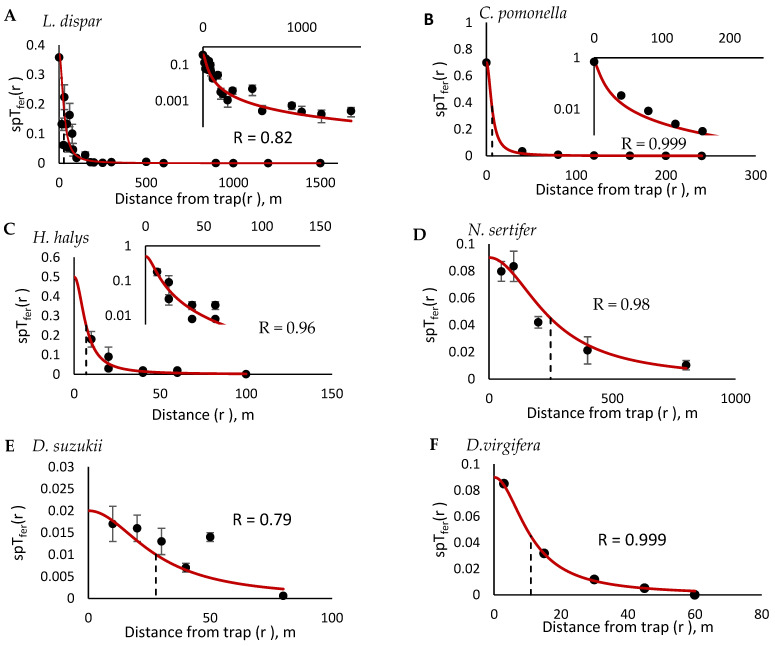

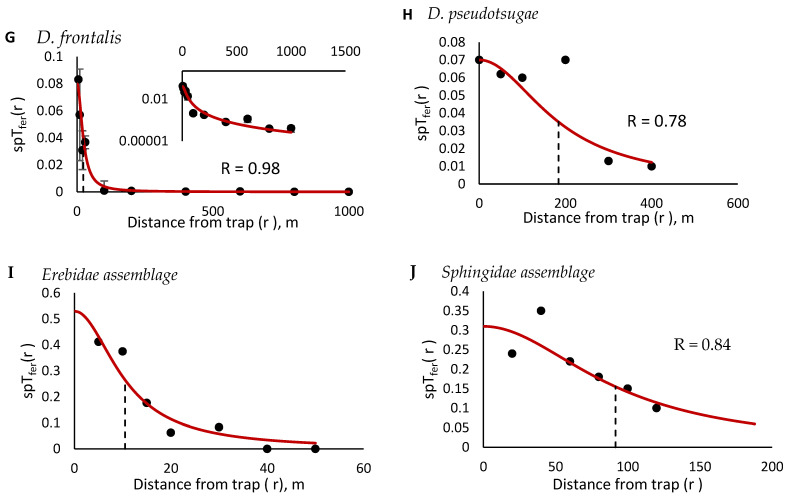
Proportion of insects caught in pheromone-baited traps placed at various distances from the release point (±SEM, where available). Black dots represent the experimental data, red lines represent the log–log model with *spT_fer_*(0) obtained using untransformed data. Black dashed lines mark *D_50_* estimated from the data as a distance, with *spT_fer_*(*r*) = 0.5*spT_fer_*(0). *L. dispar* (**A**), *C. pomonella* (**B**), *H. halys* (**C**), *N. sertifer* (**D**), *D. suzukii* (**E**), *D.virgifera* (**F**), *D. frontalis* (**G**), *D. pseudotsugae* (**H**), *Erebidae assemblage* (**I**), *Sphingidae assemblage* (**J**). For *L. dispar* (**A**), *C. pomonella* (**B**), *H. halys* (**C**), and *D. frontalis* (**G**), the insets show the fit in logarithmic scale on the y-axis, *ln(spT_fer_*(*r*)), to better illustrate the behavior at large distances from the trap, where trap catches are very low.

We note that the probability of catching an insect located in the immediate proximity to the trap, *spT_fer_*(0), provides a reference point for the rest of the trap catches. This is one reason why it is important to measure *spT_fer_*(0) empirically, since, as we saw in the example based on the *L. dispar* data, while estimating *spT_fer_*(0) by fitting Equation (2) to the experimental data is possible, the result may not always match the experimentally obtained *spT_fer_*(0), which, in turn, may lead to an over- or underestimated *D_50_*. In western corn rootworm (see Results), our estimated *D_50_ =* 11 m agrees with the results reported by Wamsley et al. [48], who observed significant drop of trap catches beyond 30 m away from the trap. However, the trap catch collected at the distance of 16 m away from the trap was also significantly lower compared to the catch in a trap located 3 m away (see Figure 2F). This discrepancy, once again, demonstrates the importance of measuring *spT_fer_*(0) empirically rather than estimating it by fitting Equation (2) to an incomplete experimental dataset. In Douglas fir beetle, *D. pseudotsugae*, previous studies reported that traps attracted beetles from at least 200 m [49], but beyond this distance, the recapture rate dropped, which agrees with our estimate of *D_50_* =184 ± 33 m (see Results).

When the proposed theory is applied to estimates of population density and related bounds in natural populations, the trapping time must equal the time to reach a converged catch for a given insect as defined by release–recapture experiments in which traps are checked daily following the release; the catch in these experiments is assumed converged when it stops increasing with increased trapping time (known times to reach a converged catch are reported in Table S2). For an insect with yet unknown values of *spT_fer_*(0) and *D_50_*, these experiments can be used to also determine them. Application in management programs is described in Onufrieva et al. [46].

## 3. Results

To validate this approach, we had to validate Equation (2). The results of the analysis conducted to estimate *spT_fer_*(0) and *D_50_* for all the insects studied here are shown in Table 1 and Figure 2.

**Figure 3:**
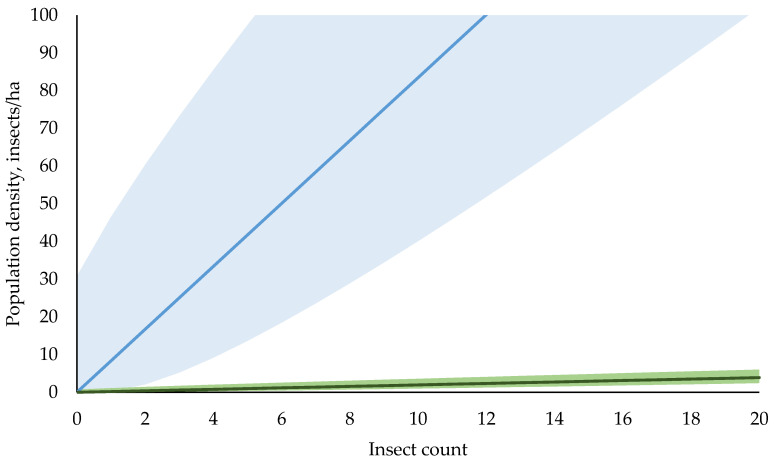
Absolute population densities as a function of catch (insect count M) in baited traps for codling moth (blue) and European sawfly (green). Light blue and green areas indicate the ranges between lower and upper bounds with 95% confidence for codling moth and European sawfly, respectively. Dark lines in the middle indicate the most probable average densities $\bar{\rho_{mp}}$. For the same number of insects caught, the corresponding population densities differ by nearly two orders of magnitude.

Once the parameters of Equation (2) are obtained, one can determine key characteristics of the actual population density in the trapping area, specifically, the most likely value for the average population density, $\bar{\rho_{mp}}$, as well as its statistical upper and lower bounds as a function of the insect count *M*, see Equations (4)–(6) in Methods. These results are exemplified for two insects in Figure 3.

## 4. Discussion

In this study, we have addressed the pressing need to estimate the absolute insect population density in the field. Since direct measurement of the absolute population density is not possible, we circumvented the problem by proposing a universal mathematical relationship that connects the absolute population density with trap catches and other experimentally measurable characteristics of an insect trap system.

The centerpiece of this approach is the universal equation that faithfully describes the relationship between the probability to catch an insect and how far the insect is from the trap. The relationship is a simple formula with only two key parameters: *spT_fer_*(0), which is the probability to catch an insect released in the immediate proximity to the trap, and *D_50_*, which we define as the distance from a baited trap at which the probability to catch an insect is one-half of the probability to catch an insect released in the immediate proximity to the trap (*spT_fer_*(0)). The strength of this definition of *D_50_* is threefold: (1) it directly corresponds to what can be measured in field experiments, (2) the concept of *D_50_* can be easily illustrated on the graph of *spT_fer_*(*r*) vs. *r*, from which the *D_50_* value can be immediately estimated, at least approximately, as the value of *r* at which *spT_fer_*(*r)* = 0.5*spT_fer_*(0) (Figure 1), and (3) the definition applies to any trap type.

To understand the biological meaning of *D_50_* and its possible relationship to insect physiology we compared D_50_ values derived from the trapping experiments with direct measurements of the insect physiological response to an appropriate attractant, where available. In *L. dispar*, we estimated *D_50_* = 26 ± 3 m (Table 1), while Elkinton et al. [55] observed wing fanning starting at a distance of 20 m from the pheromone source, which agrees with our estimate of *D_50_*. Our estimate of *D_50_* for European sawfly (*D_50_* = 250 ± 21 m) agrees with the results of behavioral studies reported by Östrand et al. [56], who observed a response in *N. sertifer* to pheromone sources located 200 m away.

In Douglas fir beetle, *D. pseudotsugae*, previous studies reported that traps attracted beetles from at least 200 m [49], but beyond this distance the recapture rate dropped, which agrees with our estimate of *D_50_* =184 ± 33 m. In western corn rootworm, our estimated *D_50_ =* 11 m agrees with the results reported by Wamsley et al. [48], who observed a significant drop of trap catches beyond 30 m away from the trap. However, the trap catch collected at the distance of 16 m away from the trap was also significantly lower compared to the catch in a trap located 3 m away (Figure 2E). This, once again, demonstrates the importance of measuring *spT_fer_*(0) empirically rather than estimating it by fitting Equation (2) to an incomplete experimental dataset.

Based on the agreement of our results with physiological studies, we suggest that the qualitative biological meaning of *D_50_* is the effective attractive distance at which the probability that the lure elicits a response from the insect is substantial. The formulation of a more quantitative relationship between *D_50_* and insect physiology will require more detailed physiological experiments than those currently available. We note in passing that for *L. dispar*, the numerical values of *D_50_* and the pheromone plume reach described by Miller et al. [45] happen to be similar, but the match is purely coincidental (possibly due to a poor fit of the Miller method to the *L. dispar* data [46]) and does not hold for most insects studied here. For most insects, the values of plume reach (a pheromone-specific concept) and *D_50_* (a universal characteristic of any trap) differ significantly. For example, in European sawfly discussed above, previous studies reported a behavioral response at a distance of 200 m from the pheromone-baited trap [56], which agrees with our estimate of *D_50_* = 250 ± 21 m and is significantly different from the previously reported plume reach of 30–50 m for this insect [45].

One of the most striking results of this study is that the same number of insects caught in a trap may result from population densities different by orders of magnitude in the field (Figure 3). The qualitative explanation is that the relationship between insect population density and the trap catch (Equations (4) and (6)) is sensitive to parameters of the trap insect system, particularly *D_50_*, and *spT_fer_*(0). For example, in European pine sawfly, *D_50_* is almost 40 times larger than that of codling moth. Therefore, the European pine sawfly trap collects insects over an area almost 1600 times larger than the codling moth trap. Even though the European pine sawfly catch probability near the trap, *spT_fer_*(0), is almost eight times lower than that of codling moth, the net effect is still two orders of magnitude greater, with more insects caught for the same population density. Conversely, the same trap catch for these two insects translates into a two-order-of-magnitude difference in the underlying population densities (Figure 3). Thus, based on a trap catch alone, one cannot make any quantitative, or even qualitative, assessment of what the actual insect population might be. The meanings of “catch zero” and “catch one” become clear only in light of the established relationship with the statistical bounds of the population density (Equation (5)). When no insects are caught in the trap, we can conclude that, even though the insects might still be present in the field, their population density cannot exceed the specific threshold (upper bound, 95% confidence, Figure 3). Likewise, if only a single insect is caught in the trap, one can conclude that the actual population density cannot, with 95% confidence, be lower than the appropriate lower bound (Figure 3).

It is remarkable that the simple Equation (2) works so well (average R = 0.91) across five orders of insects collected using very different attractants, such as a chemical and light, selected randomly from the literature based on the available data, despite the fact that the parameters of the analyzed trap insect models varied widely: *D_50_* ranged from 6.5 to 250 m, and the estimated probability of catch in the immediate proximity to the trap *spT_fer_*(0) ranged from 0.02 to 0.7 (Table 1). This universality is the consequence of the universal set of principles applicable to trapping of all insects: two-dimensional active movement space (insects following the terrain), finite active life span, and converged trap catches (collection time is just long enough) used in well-designed trapping experiments.

The value of the proposed approach is that it reduces the complexity of insect behavior with respect to traps and attractants to only a few parameters to be used in the single equation universal to all insects. We stress that these parameters differ significantly between different insect trap systems. The parameters may need to be adjusted if the experimental conditions under which they were originally tabulated change significantly. For example, the parameters reported here for *L. dispar* were estimated in Virginia using USDA milk-carton pheromone-baited traps. Those parameters might be different if the same traps were deployed in Wisconsin or if a Delta-style trap was used in Virginia instead of the USDA milk-carton trap.

We note that even though the main prediction–the absolute population density-cannot be measured directly, it can still be validated indirectly. Namely, if the quantity that needs validation is connected precisely to another characteristic of a system that can be experimentally checked, then validating the latter is just as good. For example, weighing a live blue whale is impossible in practice; however, current digital photography permits an accurate reconstruction of an animal’s 3D image and, hence, its volume V. From that, the mass of the animal can be calculated exactly using m = ρV (ρ of a blue whale = ρ of H_2_O). In this trivial example, experimental verification of a prediction of m is equivalent to validating the prediction of V.

The importance of conservation and pest management programs cannot be overstated as climate change, loss of biodiversity, and biological invasions remain the most serious environmental problems facing society. The inability to interpret insect trap catch data quantitatively, which includes directly relating trap catches to the absolute population density of an insect, hinders conservation, management, and research programs by making it difficult to provide recommendations, develop management tactics, and evaluate treatment efficacies. The universal method reported here fills a key knowledge gap: it allows a rigorous estimation of the most likely insect population density, along with the corresponding upper and lower bounds, from the number of insects caught by a single trap. We emphasize that the experimental measurement of the absolute density of a native population in the field is completely out of reach in practice, and so the only way to estimate the population density is to relate it, via a mathematical method, to characteristics of the population that can be measured. The method we are proposing is universal, in that it can be used for any trap insect system. We believe this method will help develop technologies for improved insect population detection and management but, most importantly, will help drive future basic and applied research in multiple areas of entomology and ecology. The proposed method, for example, might allow to directly compare the efficacy of multiple traps used for the same insect.

Step-by-step instructions along with supporting files and scripts for using the proposed method are included in the Supplementary Materials.

## Figures and Tables

**Table 1:** Estimates of the probability to catch an insect released in the immediate proximity to the trap (*spT_fer_*(0)) and *D_50_* for various insects in the orders Lepidoptera, Coleoptera, Hymenoptera, Diptera, and Hemiptera. Experimental R_max_ is listed for *L. dispar*, *N. sertifer*, *D. frontalis*, *D. pseudotsugae*, and *Erebidae* assemblage; for *C. pomonella*, *H. halys*, *D. suzukii*, *D. virgifera*, and *Sphingidae* assemblage, R_max_ was estimated using the method described by Miller et al. [45] and is marked with an *.

Insect	Trap Type	*spT_fer_*(0)	*D_50_* ± SEM, m	*R_max_*, m
*L. dispar*	Pheromone	0.37	27.3 ± 3	1600
Codling moth (*C. pomonella*)	Pheromone	0.7	6.5 ± 1	260 *
Brown marmorated stink bug (*H. halys*)	Pheromone	0.5	7 ± 0.9	130 *
European pine sawfly (*N. sertifer*)	Pheromone	0.09	250 ± 21	1040
Spotted wing drosophila (*D. suzukii*)	Chemical	0.02	27.7 ± 7	90 *
Western corn rootworm (*D. virgifera*)	Pheromone	0.09	11 ± 0.4	60 *
Southern pine beetle (*D. frontalis*)	Pheromone	0.08	23 ± 2.8	1000
Douglas fir beetle (*D. pseudotsugae*)	Pheromone	0.07	184 ± 33	600
Erebidae assemblage	Light	0.52	10.6 ± 1.4	40
Sphingidae assemblage	Light	0.31	91.6 ± 8	175 *

## Data Availability

To develop the methodology, we used previously published data that are available in the respective publications cited in this paper. The files for conducting the analysis are available at https://doi.org/10.7294/BE34-ZS61 (accessed on 10 October 2021).

## Supplementary Materials

The following are available online at https://www.mdpi.com/article/10.3390/insects12100932/s1, Step-by step instructions for using the proposed method, Table S1: Quantile function of the χ^2^ distribution with n degrees of freedom, *p* = 0.95, to be used in Equation (5)., Table S2: Time to converged catch obtained using release-recapture experiments.
