# Supplementary material for: How to Count Bugs: A Method to Estimate the Most Probable Absolute Population Density and Its Statistical Bounds from a Single Trap Catch
Source: Insects. 2021 Oct 13;12(10):932. doi: 10.3390/insects12100932 (PMC8540812; doi:10.3390/insects12100932)
Supplement: Supplementary file 1 [file insects-12-00932-s001.zip › insects-1386971-supplementary.pdf]

Supplementary Material

Supplementary files and programs are available for download from https://doi.org/10.7294/BE34-ZS61 (accessed on October 20, 2021)

TrappingData.xls (Microsoft Office 2019) contains 2 sheets: "Data table" (for entering experimental trap catch data) and "Absolute density" (for calculating most probable absolute density and its bounds associated with various trap catches).

Script *Tfer0.jsl* (JMP® Pro 16, SAS Institute, 2020) is used to estimate trap catch in the immediate proximity to a trap ($spT_{fer}(0)$) by fitting the model to the available trap data points at larger distances from the trap; however, we strongly encourage empirical measurement of this important parameter.

Script "*AbsoluteDensity.jsl*" (JMP® Pro 16, SAS Institute, 2020) is used to calculate D_{50} from the experimental trapping data.

To run the scripts, all 3 files (*TrappingData.xls*, *Tfer0.jsl*, and *AbsoluteDensity.jsl*) need to be located in the same folder. Two likely usage scenarios are described below.

The units are: D_{50} and R_{max} in meters, absolute population density in number of insects per ha.

Step by step instructions to calculating most probable absolute population density and its 95% confidence bounds.

Scenario 1: Insect-trap system with known parameters $spT_{fer}(0)$, D_{50} and R_{max}.

1. When the parameters of the insect-trap system are known, only *TrappingData.xls* is needed, see the above link.

   As an example, we will use the parameters obtained for European pine sawfly, *N. sertifer*:

$spT_{fer}(0) = 0.09$

$D_{50} = 250$

$R_{max} = 1040$

2. Plug these parameters in the corresponding cells in sheet "Absolute density" replacing values currently there. This will automatically update values of μ, lower and upper bounds, and most probable catches. The graph will also automatically update to reflect these changes.

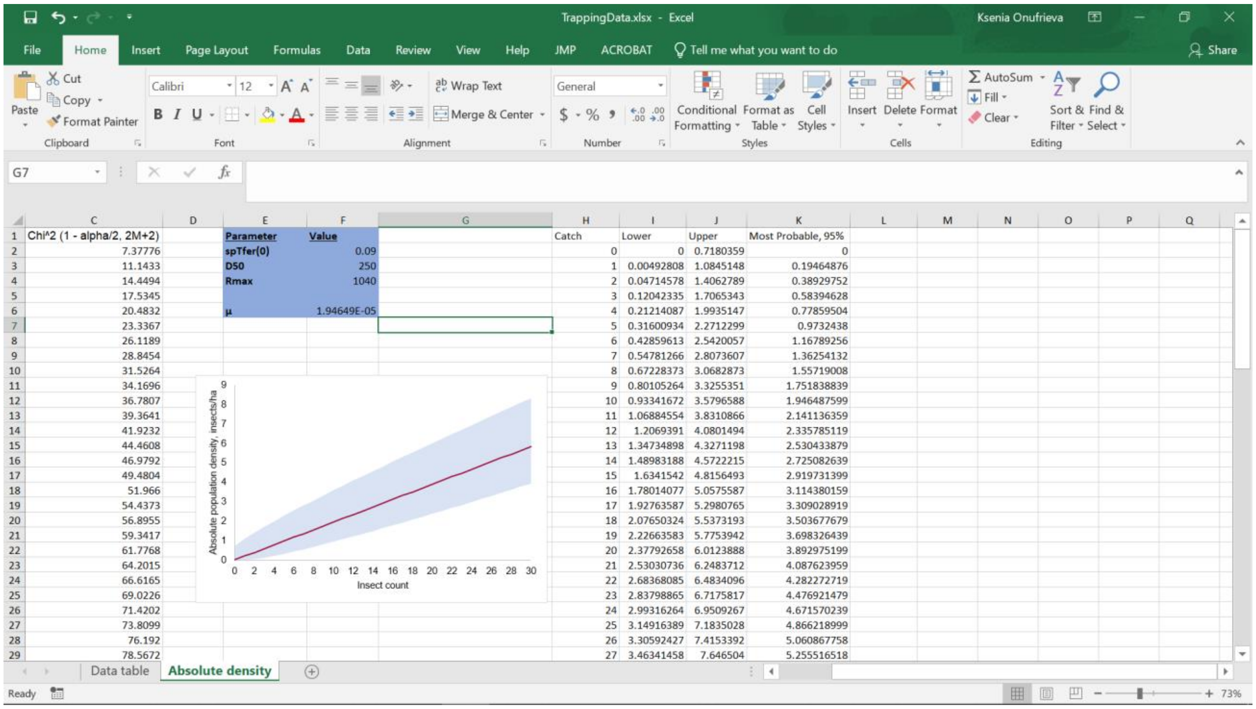

3. This graph can now be used to analyze future field data. Suppose 1 insect was caught in a trap over the converged catch period for this insect. The graph and the table can be used to estimate absolute population density for the given insect – in this case 1 insect means that absolute population density ranges 0.005 – 1.08 insects/ha, most probably 0.2 insects/ha.

Scenario 2: Insect-trap systems with unknown parameters $spT_{fer}(0)$ and/or D_{50}

1. Conduct release-recapture experiments to estimate recapture rates at ≥ 5 distances, including 0 m (in the immediate proximity to the trap, which is *$spT_{fer}(0)$*) and large distances to approximate *R_{max}* (smallest distance at which trap catch is 0). Catch should correspond to converged catch (minimum number of days N after which trap catch stops increasing). The same N needs to be used in the field experiments designed to sample wild populations.

2. Download *TrappingData.xls* and two JMP scripts, *Tfer0.jsl* and *AbsoluteDensity.jsl*, needed to perform a fit to estimate parameters (*$spT_{fer}(0)$* and *D_{50}*) from experimental data.

3. Enter trap catch data in the sheet "Data table" replacing the data that are already there, do not change the column headers.

4. If *$spT_{fer}(0)$* is missing and cannot be obtained empirically, it can be estimated using *Tfer0.jsl* script (JMP® Pro 15, SAS Institute, 2019), but it is not recommended.

5. To conduct the analysis, please, make sure that all files are located in the same folder. As an example, we will use data that is already entered in the spreadsheet "Data table".

6. If the *$spT_{fer}(0)$* data is missing, we will estimate it using the script *$T_{fer}0$.jsl*. However, we do recommend collecting this data empirically. To estimate *$spT_{fer}(0)$* using the script:

   a. Double-click the script *$T_{fer}0$.jsl*, this will open the window:

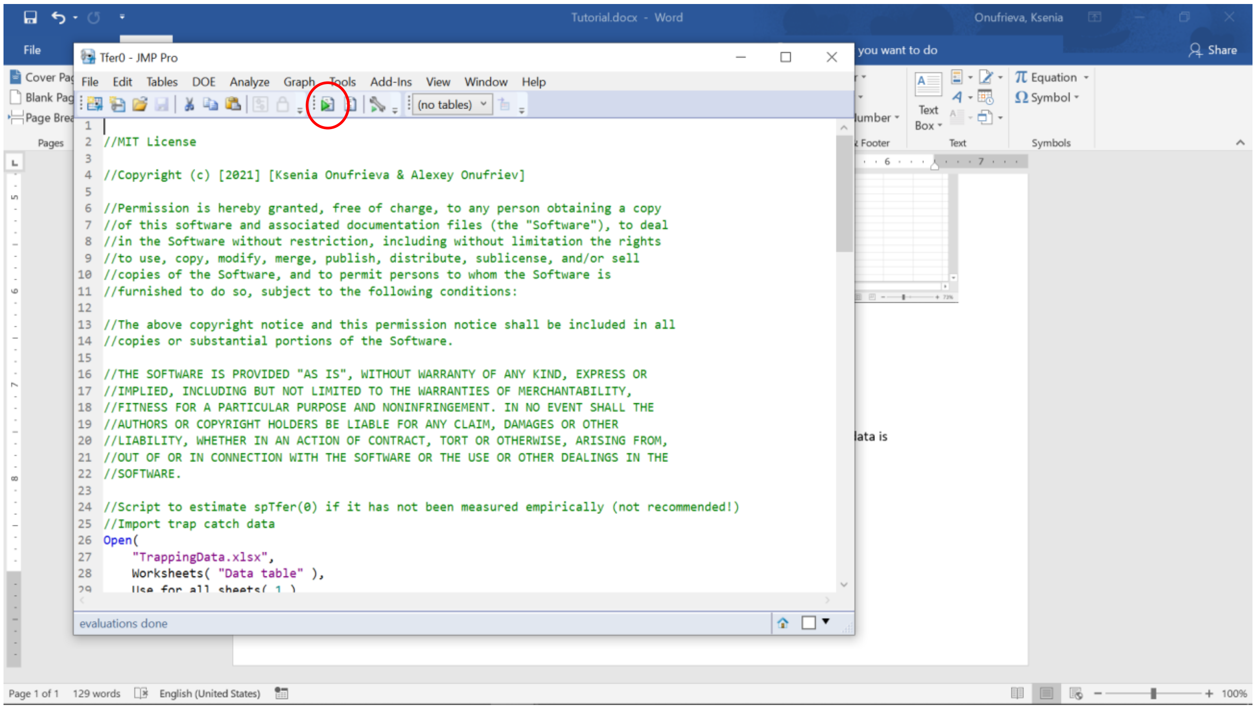

b. Click Run Script (shown by red circle in the picture above), $spT_{fer}(0)$ will be calculated and reported in the table below (circled in red).

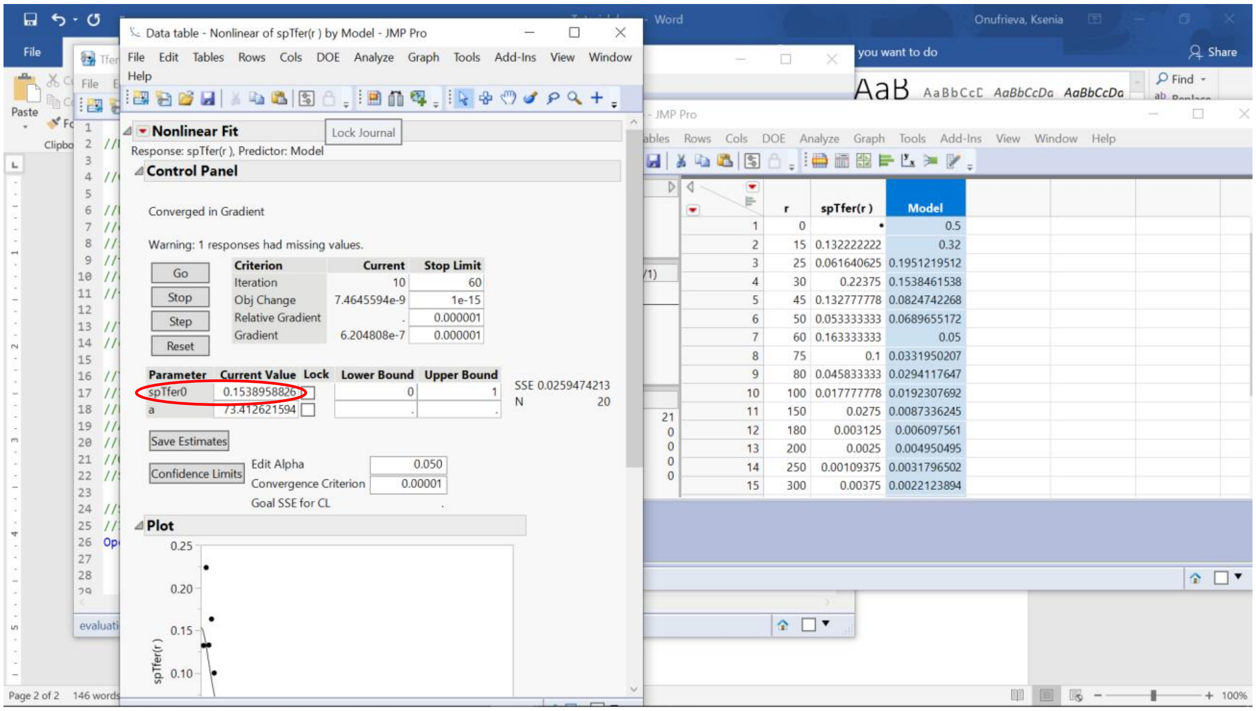

c. Add the value of $spT_{fer}(0)$ to the corresponding cell in sheet “Data table” of *TrappingData.xls* file. Now the data table is ready to use for estimating D_{50} from the data.

7. To estimate D_{50} by fitting Equation 3 to the log-transformed experimental data points:

   a. Double-click the script AbsoluteDensity.jsl, this will open the window

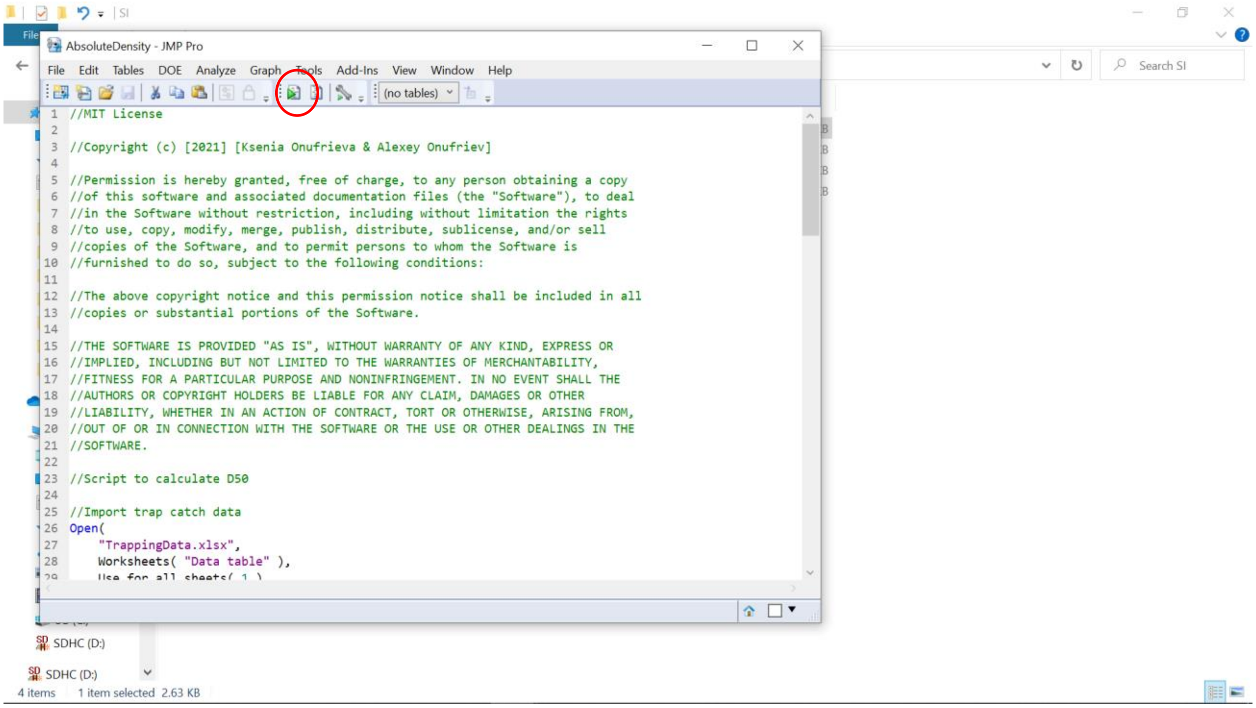

   b. Click Run Script (circled in red in the figure above), this will calculate D_{50} (circled in red below)

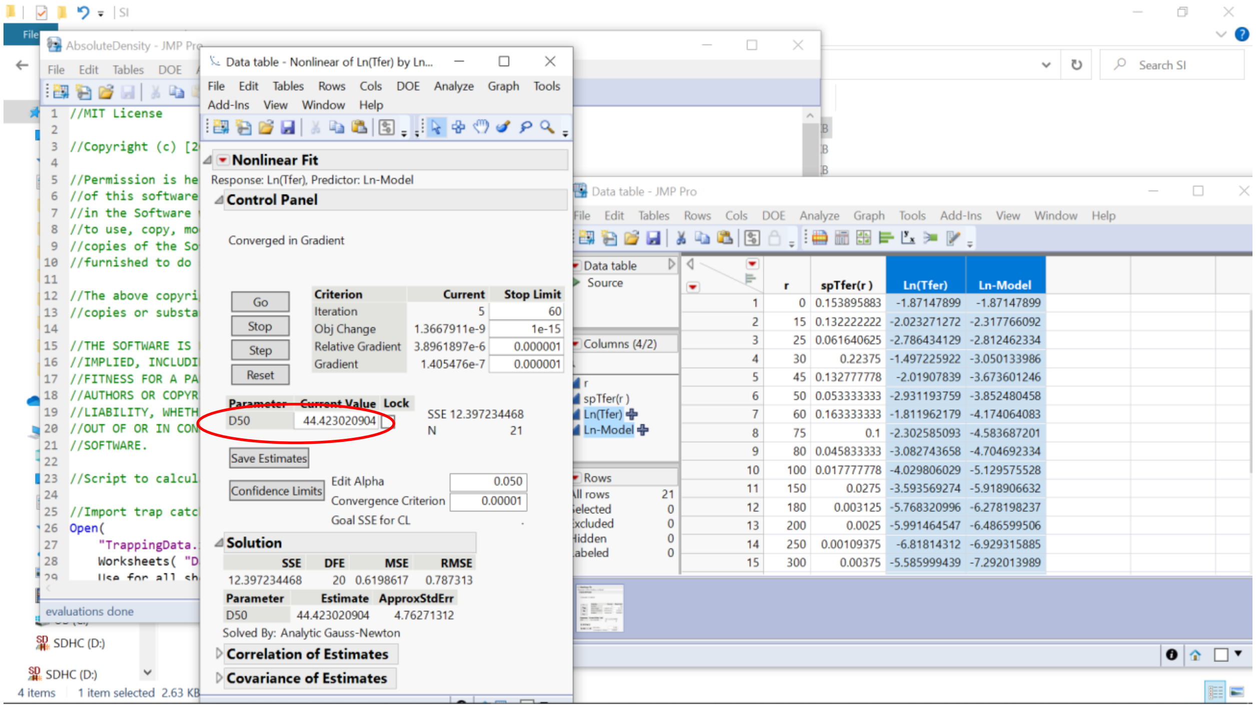

   c. Plug both parameters, $spT_{fer}(0)$ and D_{50}, in the corresponding cells in spreadsheet "Absolute density" replacing values that are currently there. This will automatically update values of μ,

lower and upper bounds, and most probable catches. The graph will also automatically update to reflect these changes.

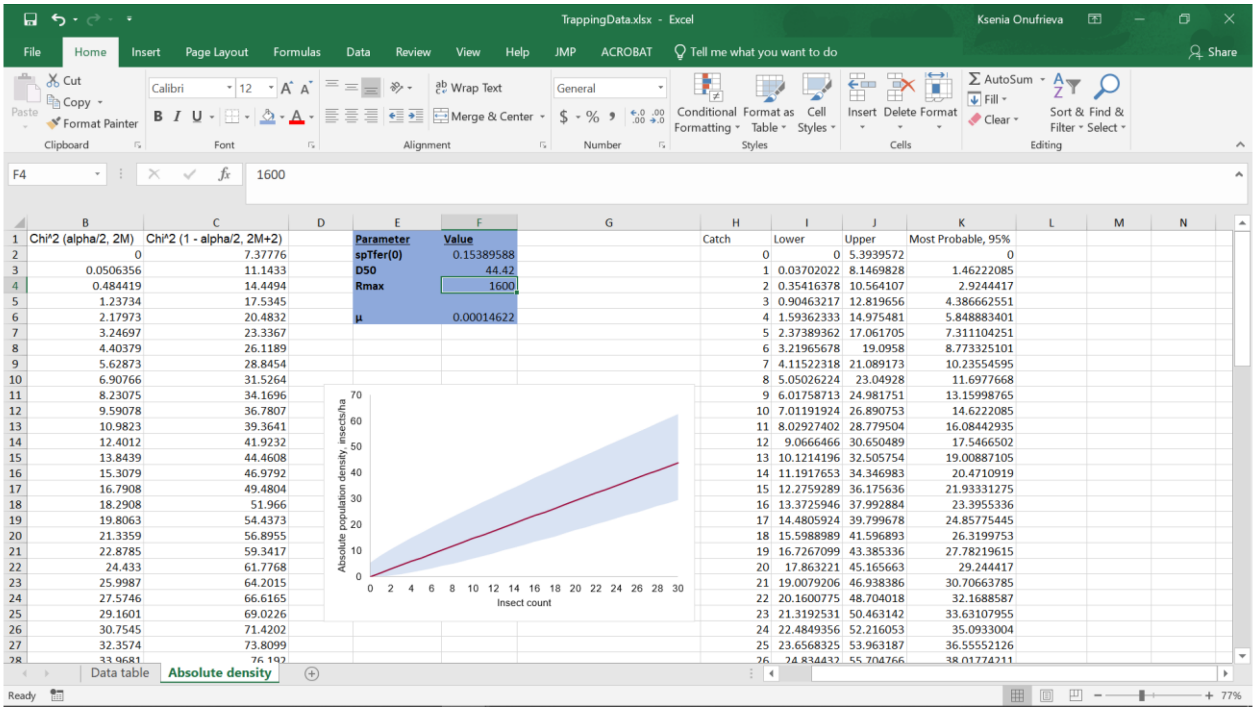

d. The graph is now ready to be used to interpret field data. Suppose 1 insect was caught in a trap over the converged catch period for this insect. The graph and the table can be used to estimate absolute population density for the given insect – in this case 1 insect means that absolute population density ranges 0.037 – 8.15 insects/ha, most probably 1.5 insects/ha.

Table S1. Quantile function of the χ^2 distribution with n degrees of freedom, p = 0.95, to be used in *Equation 5*. Alternatively, Excel table and scripts available at https://doi.org/10.7294/BE34-ZS61 (accessed on October 10, 2021) can be used to calculate most probable absolute population density and its statistical bounds.

# males caught	χ^2 ((1 – p)/2, 2M)	χ^2 (1 – (1 – p) /2, 2M+2)
0	0	7.37776
1	0.0506356	11.1433
2	0.484419	14.4494
3	1.23734	17.5345
4	2.17973	20.4832
5	3.24697	23.3367
6	4.40379	26.1189
7	5.62873	28.8454
8	6.90766	31.5264
9	8.23075	34.1696
10	9.59078	36.7807
11	10.9823	39.3641
12	12.4012	41.9232
13	13.8439	44.4608
14	15.3079	46.9792

15	16.7908	49.4804
16	18.2908	51.966
17	19.8063	54.4373
18	21.3359	56.8955
19	22.8785	59.3417
20	24.433	61.7768
21	25.9987	64.2015
22	27.5746	66.6165
23	29.1601	69.0226
24	30.7545	71.4202
25	32.3574	73.8099
26	33.9681	76.192
27	35.5863	78.5672
28	37.2116	80.9356
29	38.8435	83.2977
30	40.4817	85.6537

Table S2. Time to converged catch obtained using release-recapture experiments.

**Insect**	**Trap type**	**Time to converged catch**
L. dispar	Pheromone	3 days
Brown marmorated stink bug (*H. halys*)	Pheromone	2 days
Erebidae assemblage	Light	10 min
Sphingidae assemblage	Light	10 min
